# Female family caregivers face a higher risk of hypertension and lowered estimated glomerular filtration rates: a cross-sectional, comparative study

**DOI:** 10.1186/s12889-015-1519-6

**Published:** 2015-02-22

**Authors:** Yasuko Torimoto-Sasai, Ayumi Igarashi, Takashi Wada, Yasuko Ogata, Noriko Yamamoto-Mitani

**Affiliations:** Graduate School of Health Care Sciences, Tokyo Medical and Dental University, 1-5-45 Yushima, Bunkyo-ku, Tokyo 113-8519 Japan; The Dia Foundation for Research on Ageing Societies, 3F, Naota, bld, 1-34-5 Shinjuku, Shinjuku-ku, Tokyo, 160-0022 Japan; Division of Health Sciences & Nursing, Graduate School of Medicine, the University of Tokyo, 7-3-1 Hongo, Bunkyo-ku, Tokyo 113-0033 Japan; Shimbashi Medical Checkup Office, the Jikei University School of Medicine, 3-25-8 Nishi-simbashi, Minato-ku, Tokyo 105-8461 Japan

**Keywords:** Family caregiving, Caregiver health, Physical health, Hypertension, Estimated glomerular filtration rate (eGFR), Japan

## Abstract

**Background:**

Despite societal efforts to alleviate the challenges, caregiving seems to constitute a substantial burden and source of stress for many families of older adults in Japan. However, precise information on the physical health of caregivers, based on objective data, is not available. The purpose of this study was to improve the understanding of the physical health of Japanese family caregivers using objective indicators and a comparative research design.

**Methods:**

A cross-sectional, comparative study was conducted among family caregivers and their non-caregiver counterparts. Surveyors visited caregivers in their homes to administer a questionnaire survey, measure their blood pressure, and collect blood samples using a kit. Blood samples were tested for LDL-Cholesterol, HDL-Cholesterol, AST, ALT, γ-GTP, uric acid, creatinine and HbA1c. Non-caregiver data were collected at a university-based health checkup center. We compared 149 caregivers with 149 sex- and age-matched non-caregivers using conditional logistic regression analyses to examine the impact of caregiving, adjusting for multiple control variables. Analyses were conducted separately for men and female.

**Results:**

The prevalence of high blood pressure was significantly higher among caregivers than non-caregivers (male: 72.7% among caregivers vs. 40.9% among non-caregivers, female: 57.1% vs. 27.6%, respectively). After adjusting for related sociodemographic and health factors, high blood pressure remained significantly more prevalent among caregivers than non-caregivers, only among female (adjusted OR=2.16, 95% CI [1.14, 4.08]). Female caregivers showed lower eGFR than their non-caregiver counterparts (adjusted OR=6.54, 95% CI [2.38, 17.91]). No significant differences were observed between the two groups on any other indicators.

**Conclusions:**

Results suggest that female caregivers are at a higher risk of conditions such as cerebral, cardiovascular or kidney diseases than non-caregivers. Steps must be taken to identify caregivers with high blood pressure and lowered eGFR and provide them with the support they need before these risk factors develop into serious diseases.

## Background

Japan is one of the fastest ageing societies in the world. As one strategy to meet the needs of a growing elderly population, the Japanese government introduced public long-term care insurance (LTCI) for the elderly in 2000. One of the aims of this new insurance system is to help older people live independently. In response to this new system, the availability of services increased rapidly, including home-based care services. The number of persons certified as needing long-term care has also increased substantially; the number of in-home services users increased from 971,461 in 2000 to 3,062,232 in 2011 [1].

Although the introduction of the LTCI initiated a transition from family caregiving to socialized caregiving, the original LTCI did not take family caregivers into account; LTCI services were provided only to care recipients based on assessments of their physical and cognitive health, without assessing family caregivers. To address this problem, in the 2006 revision of the LTCI, the Japanese government encouraged each municipal government responsible for managing the LTCI to handle any support programs for family caregivers, such as counseling services. In practice, however, the programs have not been widely implemented, partly because they were not mandatory. As such, support for family caregivers has not been as advanced.

Many previous studies have reported that most family caregivers today feel the burden of caregiving and often report their own health problems, such as psychiatric symptoms or other complaints [2-11]. However, the assessment of caregivers’ health in these studies has been largely subjective, rather than collecting objective data. Furthermore, one study reports that many female caregivers do not have time to visit a physician for a checkup [12]. In these cases, it is impossible to evaluate the family caregivers’ health, even though they might be prospective care recipients in future.

Developing an understanding of the objective health of caregivers and the impact that caregiving has on their health is of primary importance. The purpose of this study was to examine the objective health of caregivers and to assess the impact of caregiving.

## Methods

**Figure 1:**
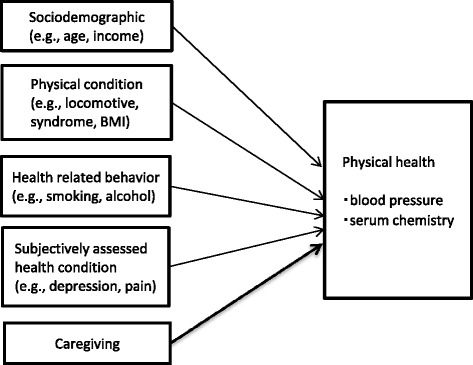
**Conceptual framework.**

A cross-sectional, comparative study based on a conceptual model (Figure 1) was conducted among family caregivers and their non-caregiver counterparts. While each individual’s health is affected by factors such as demographics, health behaviors, and his or her own physical or mental health conditions, we also assumed that caregiving has a significant association on the health of the caregiver. Therefore, we compared health outcome data between family caregivers and non-caregivers counterparts, while adjusting for a variety of other possible factors affecting health outcomes.

### Participants

“Caregiver” was defined as a main person taking care of an elderly family member who uses any home service under the LTCI system. Family caregivers were recruited into this study through 26 agencies providing LTCI services, such as care management, home care nursing or home help, in the urban areas of Tokyo, Osaka, Kobe, and Ibaraki.

We also recruited non-caregivers who were not providing care at the time of the survey. Non-caregivers were recruited through a university health checkup center located in an urban area in Tokyo. The center provided preventive health examinations; it did not provide any medical treatment, but could refer patients directly to the university hospital if any abnormal results were found. In both groups, additional inclusion criteria were the absence of any cognitive disorders and the ability to read and write Japanese without assistance.

### Data collection procedure

We collected data on caregivers and non-caregivers using different procedures. Caregiver data were collected in participants’ homes by surveyors with nursing licenses, because caregivers have minimal time to spare to travel to a study site. First, the surveyors called potential participants to confirm their intention to participate in the study. During their visit, surveyors administered a questionnaire survey, then took a blood pressure reading and collected a blood sample using a kit [13]. We trained 13 registered nurses in a half-day training session, teaching them the rules and practical skills needed for the survey, as well as the standardized procedure for conducting blood pressure measurements and blood sampling using the kit. Data from non-caregivers were collected at the health checkup center. Non-caregiver questionnaires were self-administered because of time and space limitations at the center. Blood pressure readings and serum chemistry data were collected from the health checkup data.

In both groups, prior to the data collection, the surveyors explained to participants their rights to participate in or leave the study, the confidentiality of personal identification, and the freedom to leave the study without any disadvantage. After the potential participants agreed to participate in the study and signed a consent form, the data collection procedure was initiated. This study was approved by the ethics committee of Jikei University. The data were collected from June 2011 to August 2012.

### Measurements

#### Questionnaires

The questionnaires consisted of questions regarding sociodemographic variables, physical condition variables, health-related behavior variables, and subjectively assessed health condition variables. Sociodemographic variables included age, sex, educational history, working status, household income, and marital status. Physical condition variables included the locomotive syndrome as measured by the Japanese Orthopedic Association method [14], weight, height, body mass index (BMI), and menopause status (female only). Locomotive syndrome was defined as lowered activities of daily living caused by disabilities of the locomotive organs and the risk of physical fragility [15]. The syndrome was assessed by five symptoms including pain, bone malformation, limitation of joint motion range, muscle loss, and loss of balance. Health-related behavior variables included alcohol consumption, smoking, and exercise. Subjectively assessed health condition variables included sleep disorder as measured by Pittsburgh Sleep Quality Index [16,17], depression as measured by the K6 [18], pain as measured by the Margolis rating system [19], and other health-related complaints [6] with history of medical treatments and medications. Information on care recipients and the caregiving situation was collected from caregivers, including sex, age, activities of daily living (ADL), instrumental activities of daily living (IADL), LTCI-certified care level, average time spent caregiving per day, or number of years of caregiving. The IADL was assessed by an instrument developed by Lawton [20]; it consists of 31 items in eight domains, such as making a phone call or going shopping. As an indicator of service use, we collected data on the amount of copayment spent for LTC services. The copayment for service use was 10% of the total cost under the LTCI.

#### Serum chemistry

The serum chemistry data used in this study were as follows: low-density lipoprotein cholesterol (LDL-C), high-density lipoprotein cholesterol (HDL-C), aspartate aminotransferase (AST), alanine aminotransferase (ALT), gamma-glutamyl transpeptidase (gamma-GTP), uric acid (UA), creatinine (Cr) and glycosylated haemoglobin (HbA1c). Each variable was recoded into a dichotomous variable indicating whether the participant was within or outside of the normal range, based on the criteria described below.

#### Criteria for normalcy

##### High blood pressure

The criterion for high blood pressure was a blood pressure of 140 mmHg and more systolic (SBP) or 90 and more diastolic (DBP), based on the guidelines of the Japanese Society of Hypertension [21]. We also included individuals taking anti-hypertensive medications (receiving medication) in the high blood pressure group.

##### eGFR

The estimated glomerular filtration rate (eGFR) is a test to evaluate chronic kidney disease (CKD), often used in clinical practice or in epidemiological studies. Lower eGFR is considered a risk factor for CKD [22]. In this study, eGFR results calculated by standard formula for Japanese [22] were dichotomized into either less than 60 mL/min/1.73 m^2^ or within the normal range. Any participant with less than 60 mL eGFR was instructed to consult a physician [22].

##### Dyslipidemia

Standard primary prevention criteria for dyslipidemia were used: less than 40 mg/dL HDL-C or 120 mg/dL and more LDL-C [23]. We also included participants being treated for dyslipidemia in the “dyslipidemia” group.

##### Liver function

The criteria for liver function were as follows: more than 30 IU/L AST, more than 30 IU/LALT, or more than 50 IU/L γ-GTP, as recommended by the Japan Society of Ningen Dock [24]. We also categorized participants being treated for liver function in the out-of-normal-range group, with the exception of viral hepatitis. We included those with viral hepatitis as normal because their lowered liver function was not due to caregiving responsibilities.

##### Hyperuricemia

The criteria for hyperuricemia were either a UA level of 7.0 mg/dL and more, as suggested by the Japanese Society of Gout and Nucleic Acid Metabolism [25], or the current treatment of hyperuricemia.

##### Diabetes

The criteria for diabetes were either an HbA1c level of 6.5% and more according to the Japan Diabetes Society [26], or the current treatment of diabetes.

### Data analyses

Since there was considerable difference between the caregiver and non-caregiver groups in terms of age and sex, we matched caregiver data to non-caregiver data based on these two characteristics for the purposes of the analyses, stratifying age into five year categories. We analyzed the male and female data separately, because previous studies have suggested biological and social sex differences in caregivers’ attitudes and the burden of caregiving, as well as the risk of the outcome variables [27].

Because the blood samples of the groups were obtained using different methods, we transformed the data in the caregivers’ sample using regression equations to adjust for systematic errors [13,28] and improve measurement reliability. Those regression equations were developed from separate blood samples from 48 persons obtained using both methods. From the data, we developed the equation y=b + ax (with y as data with the intravenous method used for non-caregivers and x as data with the kit method used for caregivers), and obtained estimates for “a” and “b.” The data of the caregivers’ blood samples (x) was thus transformed and the values obtained as y were used.

We first conducted bivariate analyses to examine the differences in sociodemographic, physical condition, health-related behavior and subjectively assessed health condition variables between caregivers and non-caregivers. Chi-square or Mann-Whitney U tests were used, depending on the type of data. Then, we conducted multivariate conditional logistic regression, using the dichotomous variables based on results of blood pressure and serum chemistry as outcome variables. In the regression model, in addition to the caregiver or not variable as an explained variable, we added the following control variables: education, household income, work, marital status, history of high blood pressure (eGFR model only), history of locomotive syndrome, BMI, menopause (only among females), smoking, alcohol consumption, exercise, sleep disorder, depression, pain, and indefinite complaints. All statistical analyses were performed using SPSS version 19.0 (Chicago, Illinois, IBM, USA).

## Results

**Figure 2:**
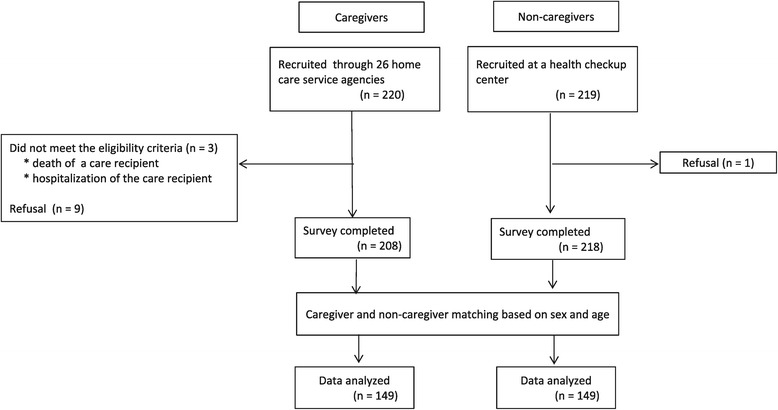
**Sampling process.**

In total, we approached 220 caregivers and 219 non-caregivers. Nine caregivers and one non-caregiver refused to participate and three caregivers were excluded due to the care recipients’ death or hospital admission at the time of our first contact; we collected data from 208 caregivers and 218 non-caregivers. From this group, we generated a dataset of 149 matched pairs of caregivers and non-caregivers; the remaining unmatched data was excluded from the analyses (Figure 2).

### Subject characteristics

For male, the mean age was 67.3 (SD=9.1) among caregivers and 67.0 (SD=9.2) among non-caregivers, respectively. For female, the mean age was 61.4 (SD=10.1 and 10.2, respectively) in both groups.

The caregivers had less education (p < 0.001 for both male and female) and less household income (p < 0.001 for both) than non-caregivers. Fewer male caregivers were working than their non-caregiver counterparts (p < 0.001) (Table 1). Male caregivers had lower BMIs than non-caregivers (p=0.027), while female caregivers had higher BMIs (p=0.037). More female caregivers had locomotive syndrome (p=0.032), pain (p < 0.001) and indefinite complaints (p=0.038) than non-caregiving female. As for the care recipients, the mean age was 81.2 (SD=10.2) for male caregivers and 82.7 (SD=9.9) for female caregivers, with 39.5% and 44.9% of care recipients being bedbound, respectively. The male and female caregivers spent an average 11.6 (SD=9.0) and 10.8 (SD=7.4) hours per day providing care and had been providing care for an average of 5.1 (SD=4.9) and 6.7 (SD=5.0) years respectively.

**Table 1:** **Sociodemographic and health characteristics of participants and care recipients**

		**Male**				**Female**			
		**All (n=88)**	**Caregivers (n=44)**	**Non-caregivers (n=44)**		**All (n=210)**	**Caregivers (n=105)**	**Non-caregivers (n=105)**	
**Participants**					**p value**				**p value**
Demographics									
Age (years)	mean ± SD	67.1 ± 9.1	67.3 ± 9.1	67.0 ± 9.2	0.907	61.4 ± 10.2	61.4 ± 10.1	61.4 ± 10.2	0.972
Education (years)	mean ± SD	13.8 ± 2.9	12.8 ± 2.7	14.8 ± 2.7	<0.001	13.4 ± 2.7	12.5 ± 2.2	14.3 ± 2.8	<0.001
Household income	<US$ 50,000; n (%)	41 (48.2)	35 (83.3)	6 (14.0)	< 0.001	108 (54.3)	73 (70.9)	35 (36.5)	< 0.001
Work status	employed; n (%)	41 (46.6)	10 (22.7)	31 (70.5)	< 0.001	81 (39.5)	35 (33.3)	46 (46.0)	0.086
Marital status	single; n (%)	19 (21.6)	15 (34.1)	4 (9.1)	0.008	61 (29.2)	30 (28.6)	31 (29.8)	0.880
Physical conditions									
Locomotive syndrome	n (%)	28 (31.8)	16 (36.4)	12 (27.3)	0.493	78 (37.1)	47 (44.8)	31 (29.5)	0.032
BMI (kg/m^2^)	mean ± SD	23.5 ± 3.2	22.8 ± 3.3	24.3 ± 2.9	0.027	22.4 ± 3.6	22.9 ± 3.7	21.9 ± 3.4	0.037
Menopause	n (%)	-	-	-	-	165 (78.9)	78 (74.3)	87 (83.7)	0.126
Health-related behaviors									
Smoking	n (%)	25 (28.4)	15 (34.1)	10 (22.7)	0.345	17 (8.1)	10 (9.5)	7 (6.7)	0.614
Alcohol	n (%)	59 (67.8)	31 (72.1)	28 (63.6)	0.493	86 (41.0)	38 (36.2)	48 (45.7)	0.206
Exercise	n (%)	37 (42.0)	17 (38.6)	20 (45.5)	0.666	68 (32.4)	26 (24.8)	42 (40.0)	0.027
Having annual health check-up	n (%)	62 (73.8)	24 (57.1)	38 (90.5)	0.001	159 (77.9)	70 (67.3)	89 (89.0)	< 0.001
Subjectively-assessed health									
Sleep disorder (Pittsburgh Sleep Quality Index)	n (%)	24 (34.3)	14 (34.1)	10 (34.5)	1.000	70 (37.4)	43 (41.7)	27 (32.1)	0.224
Depression (K6)	n (%)	5 (5.9)	1 (2.3)	4 (9.8)	0.192	10 (4.8)	7 (6.7)	3 (2.9)	0.332
Pain (Margolis rating system)									
(score range 0–45)	mean ± SD	11.7 ± 6.1	12.6 ± 7.1	10.7 ± 4.7	0.164	14.2 ± 7.8	15.9 ± 8.8	12.4 ± 6.3	<0.001
Indefinite complaint	> 4 syndromes; n (%)	29 (33.0)	16 (36.4)	13 (29.5)	0.651	102 (48.8)	59 (56.2)	43 (41.3)	0.038
Blood pressure									
Systolic blood pressure (mmHg)	mean ± SD	135.5 ± 17.4	144.1 ± 15.3	127.0 ± 15.2	<0.001	128.8 ± 20.0	137.7 ± 20.3	119.8 ± 15.2	<0.001
Diastolic blood pressure (mmHg)	mean ± SD	81.5 ± 12.6	86.4 ± 12.9	76.6 ± 10.3	<0.001	77.9 ± 11.9	82.5 ± 12.4	73.4 ± 9.4	<0.001
Serum chemistry									
Creatinine (mg/dl)	mean ± SD	0.9 ± 0.2	0.9 ± 0.2	0.9 ± 0.2	0.725	0.7 ± 0.1	0.8 ± 0.1	0.7 ± 0.1	<0.001
Low-density lipoprotein cholesterol (mg/dL)	mean ± SD	112.2 ± 21.6	108.7 ± 20.5	115.7 ± 22.3	0.156	120.2 ± 29.4	119.4 ± 27.7	121.0 ± 31.1	0.370
High-density lipoprotein cholesterol (mg/dL)	mean ± SD	59.8 ± 15.6	64.7 ± 18.1	54.9 ± 10.8	0.014	75.3 ± 17.7	74.8 ± 16.8	75.9 ± 18.5	0.685
Aspartate aminotransferase (IU/L)	mean ± SD	23.1 ± 5.9	23.2 ± 4.3	23.1 ± 7.3	0.372	22.4 ± 7.0	22.3 ± 6.1	22.5 ± 7.7	0.782
Alanine aminotransferase (IU/L)	mean ± SD	21.7 ± 12.5	19.2 ± 9.0	24.3 ± 14.9	0.105	18.2 ± 13.0	16.6 ± 11.9	19.8 ± 13.9	0.004
Gamma-glutamyl transpeptidase (IU/L)	mean ± SD	41.2 ± 57.8	37.7 ± 73.7	44.6 ± 36.0	<0.001	23.6 ± 20.8	19.2 ± 19.7	28.0 ± 21.0	<0.001
Uric acid (mg/dL)	mean ± SD	5.7 ± 1.5	5.0 ± 0.2	6.3 ± 1.2	<0.001	4.6 ± 1.2	4.3 ± 1.2	4.9 ± 1.1	<0.001
Glycosylated hemoglobin (%)	mean ± SD	5.5 ± 0.7	5.4 ± 0.8	5.5 ± 0.6	0.111	5.3 ± 0.6	5.3 ± 0.7	5.3 ± 0.4	0.055
**Care recipients**									
Female	n (%)		42 (95.5)				56 (53.3)		<0.001
Age (years)	mean ± SD		81.2 ± 10.2				82.7 ± 9.9		0.430
ADL score (Barthel Index score)									
(score range 0–100)	mean ± SD		34.1 ± 31.4				32.5 ± 30.6		0.687
IADL score; male (score range 0–5)	mean ± SD		0.0				0.9 ± 1.3		0.280
female (score range 0–8)	mean ± SD		1.1 ± 1.5				0.8 ± 1.5		0.170
Certified care level 5 (bed-bound)	n (%)		17 (39.5)				44 (44.9)		0.585
Hours per day caregiving	mean ± SD		11.6 ± 9.0				10.8 ± 7.4		0.883
Duration of caregiving (years)	mean ± SD		5.1 ± 4.9				6.7 ± 5.0		0.057
Copayment of formal caregiving services use under LTCI per month (US$)	mean ± SD		186.1 ± 90.9				209.7 ± 106.9		0.194
Usage rate to the certified maximum allowable services on each care level (%)	mean ± SD		61.2 ± 25.2				65.6 ± 28.4		0.265

SD: standard deviation, BMI: body mass index, ADL: activities of daily living, IADL: instrumental activities of daily living.

Percentages for each item were calculated after excluding missing values.

The average copayment per month for service use under LTCI was USD $186.10 (SD=$90.90) for the elderly males receiving services, and USD $209.70 (SD=$106.90) for those females receiving services (p=0.194) (Table 1). The percentage of the used amount of the certified maximum allowable official services was 61.2% (SD=25.2) for male caregivers and 65.6% (SD=28.4) for female caregivers (p=0.265) (Table1).

### Physical health based on blood pressure and serum chemistry

Among male, significantly more caregivers had high blood pressure than non-caregivers (p=0.005); however, fewer caregivers had dyslipidemia (p=0.032) and liver function disorder (p=0.002) than their non-caregiving counterparts. Among female, the rates of those with high blood pressure (p < 0.001) and those with lowered eGFR (p < 0.001) were significantly higher in caregivers than in non-caregivers; however, there were significantly fewer female caregivers with dyslipidemia than female non-caregivers (p=0.002) (Table 2).

**Table 2:** **Caregiver versus non-caregiver physical health status based on blood pressure and serum chemistry**

	**Male**					**Female**		
	**All**	**Caregivers**	**Non-caregivers**		**All**	**Caregivers**	**Non-caregivers**	
	**(n=88)**	**(n=44)**	**(n=44)**		**(n=210)**	**(n=105)**	**(n=105)**	
	**n (%)**	**n (%)**	**n (%)**	**p value**	**n (%)**	**n (%)**	**n (%)**	**p value**
	[n*]	[n*]	[n*]		[n*]	[n*]	[n*]	
High blood pressure (a)	50 (56.8)	32 (72.7)	18 (40.9)	0.005	89 (42.4)	60 (57.1)	29 (27.6)	< 0.001
[receiving medication *]	[28]	[13]	[15]		[46]	[22]	[24]	
eGFR < 60 (b)	13 (14.8)	7 (15.9)	6 (13.6)	1.000	60 (28.6)	50 (47.6)	10 (9.5)	< 0.001
Dyslipidemia (c)	41 (46.6)	15 (34.1)	26 (59.1)	0.032	123 (58.6)	50 (47.6)	73 (69.5)	0.002
[receiving medication *]	[12]	[2]	[10]		[41]	[11]	[30]	
Liver function disorder (d)	31 (35.2)	8 (18.2)	23 (52.3)	0.002	47 (22.4)	20 (19.0)	27 (25.7)	0.321
[receiving medication *]	[9]	[0]	[9]		[8]	[1]	[7]	
Hyperuricemia (e)	21 (23.9)	8 (18.2)	13 (29.5)	0.317	6 (2.9)	2 (1.9)	4 (3.8)	0.683
[receiving medication *]	[7]	[4]	[3]		[0]	[0]	[0]	
Hyperglycemia (f)	17 (19.3)	6 (13.6)	11 (25.0)	0.280	11 (5.2)	3 (2.9)	8 (7.6)	0.214
[receiving medication *]	[15]	[4]	[11]		[11]	[3]	[8]	

(a) Systolic blood pressure (SBP) 140 and more or diastolic blood pressure (DBP) 90 and more or receiving medication.

(b) Less than 60 mL/min/1.73 m^2.^

(c) Less than 40 mg/dL HDL-C or 120 mg/dL LDL-C and more or receiving medication.

(d) More than 30 IU/L AST or more than 30 IU/L ALT or more than 50 IU/L γ-GTP or receiving medication with the exception of viral hepatitis.

(e) UA 7.0 mg/dL and more or receiving medication.

(f) HbA1c 6.5% and more or receiving medication.

* The number of persons receiving medication was included in the total number of persons above.

### Multivariate analyses

We conducted conditional logistic regression analyses, controlling for sociodemographic characteristics and health-related variable (see Statistical analyses section) with forced entry method. Among females, significantly more caregivers had high blood pressure (adjusted OR=2.16, 95% CI [1.14, 4.08]) and lowered eGFR (adjusted OR=6.54, 95% CI [2.38, 17.91]) than the non-caregivers (Table 3), even when controlling for other variables. Among males, there was no difference between caregivers and non-caregivers in any conditional logistic regression model.

**Table 3:** **Conditional logistic regression analysis of the impact of caregiving on health**

	**Male**	**Female**
	**Adjusted** ^**b**^ **OR**	**Adjusted** ^**b**^ **OR**
**Outcome variables** ^**a**^	**(95% CI)**	**(95% CI)**
High blood pressure	1.27	2.16
	(0.34, 4.66)	(1.14, 4.08)
eGFR < 60	0.50	6.54
	(0.02, 16.68)	(2.38, 17.91)
Dyslipidemia	1.04	0.75
	(0.26, 4.21)	(0.44, 1.27)
Liver function disorder	0.5	0.58
	(0.07, 3.34)	(0.26, 1.29)
Hyperuricemia	0.92	0.39
	(0.08, 9.99)	(0.03, 5.99)
Hyperglycemia	2.67	1.10
	(0.16, 45.34)	(0.06, 19.97)

eGFR: estimated glomerular filtration rate; OR: Odd Ratio; CI: Confidence Interval; non-caregivers were the reference group.

ORs and 95% CIs were calculated using conditional logistic regression analysis.

^a^For all analyses, caregiver1.

^b^Adjusted for years’ education, household income, work status, marital status, a history of locomotive syndrome, body mass index, menopause (only among females), smoking, alcohol, exercise, sleep disorder, depression (K6), pain, indefinite complaints, and a history of high blood pressure (only for eGFR)

## Discussion

In this study, we investigated the physical health of family caregivers using objective indicators. The results suggested that female caregivers had a higher prevalence of high blood pressure and low eGFR than non-caregiving females. To the best of our knowledge, this is one of the first studies to examine the objective health of family caregivers in Japan.

Caregiving was a significant factor in high blood pressure among females, even after adjusting for multiple related variables., For the first time in Japan, this study has shown that caregiving has a significant association with the health of caregivers, even taking other variables into account [12,29]. Stress stimulates the sympathetic nervous system and increases blood pressure [30,31]. The present findings suggest that the stress experienced by female caregivers is sufficiently severe to result in a significant increase in the number of cases of high blood pressure.

Caregiving was also significantly associated with lowered eGFR. A previous study showed a decline in eGFR following major caregiving transitions [32], which is somewhat similar to our finding. However, that study showed a longitudinal change in eGFR, not a direct difference between caregiver and non-caregiver groups. This study adds to those findings from a between-group perspective. The finding that high blood pressure due to caregiving stress leads to organic changes and a decline in kidney function (i.e., lowered eGFR) is an important result. As this condition progresses, it could lead to pathological conditions such as chronic kidney disease (CKD) [21,22]. Thus, this study suggests that caregiving stress among female family caregivers may be significant enough to lead to a functional decline of the kidneys, as well as high blood pressure. Similarly, other research has suggested that stress may also increase the risk of cerebral, cardiovascular, and kidney diseases [33-35].

Among male caregivers, significant differences were observed in the prevalence of high blood pressure but not lower eGFR, and the prevalence of high blood pressure among male caregivers was substantially higher than Japanese national rates [36]. After adjusting for control variables, however, there were no significant group differences. Further examination is needed with a more representative sample, because our male sample size was relatively small despite our best efforts to sample male caregivers. Previous reports have suggested a heightened risk of high blood pressure [29] and a decline in eGFR [32] among both male and female caregivers.

In this study, although female caregivers used the same amount of LTCI services as male caregivers, they had more healthcare problems than non-caregivers; male caregivers and non-caregivers did not show this difference. Past research has demonstrated that male caregivers report less burden than their female counterparts [37], even when caring for older relatives with more severe conditions [38]; husband caregivers tend to rate care more positively than wife caregivers, using the coping strategy of willing commitment [9]. In Japanese society, females are traditionally expected to accept the family caregiver role; as a result, female caregivers may be more burdened, receive less support from professional caregivers, or feel they are taking on the caregiving role against their will [7] despite social service use. The additional burden and stress posed by these situations may compromise their health. Further research is needed to clarify the gender differences in the impact of caregiving on health conditions (especially high blood pressure and eGFR), especially among Japanese caregivers.

Other than high blood pressure and eGFR, no significant differences were observed between caregivers and non-caregivers in dyslipidemia, liver function disorder, hyperuricemia, or hyperglycemia in either gender. A previous study showed that caregiving did not have a significant association with dyslipidemia or hyperglycemia [12], but there have been no studies examining the association between caregiving and liver function disorder or hyperuricemia.

Concerning the caregivers’ use of health services, the previous study reported female caregivers had no time for physical health check-ups [12]. In this study, the caregivers had fewer annual health check-ups than non-caregivers (Table 1). In addition to the existing LTCI services aimed at reducing caregiver burden, additional services are needed for caregivers, especially female caregivers, in order to prevent disease and to improve their health. For example, special arrangements may be necessary for them to receive health check-ups.

To clarify the results in this study, these indicators should be examined based on caregiving in a large representative sample rather than comparing caregivers to non-caregivers as two homogeneous groups. Moreover, further studies should examine additional health indicators such as ambulatory blood pressure monitoring and nutrition intake over time to better understand the causal association of caregiving on physical health.

### Limitations

Our caregiver subjects were recruited via long-term care service agencies; they may be in better health than those who do not use such services. Non-caregivers used health check-ups at the university hospital and were probably of higher socio-economic status than the general public, which might have led to better health. Moreover, the non-caregiver subjects had significantly more education, higher rates of employment and marriage, and higher income than caregivers. Thus, we may have overestimated the difference between the two groups.

In order to pay due attention to these differences, we not only controlled for the socioeconomic status (SES) in the final analyses, we attempted a variety of analytic techniques (i.e., stratified analyses according to income, conditional logistic regression analyses using incremental and decremental methods, and propensity scores). We obtained almost identical results. We compared the rates of those with high blood pressure to national data, and the caregivers had higher rates than general public for almost every stratified generation [36]. With these analyses, we concluded that the caregivers’ blood pressure and eGFR were different from non-caregivers. Further examination using community representative samples should be conducted.

Another limitation was the use of a kit for collecting blood samples from caregivers versus intravenously drawn blood from non-caregivers. Despite the statistical adjustment of the values in caregiver data, we might not have been able to correct different calibrations accurately. Finally, since this study was conducted by cross-sectional survey, we could not confirm causal effect; we need further studies to clarify causal effects of caregiving on caregiver health.

Despite these limitations, this study is one of the first attempts to evaluate objective health conditions among Japanese family caregivers who tend not to attend such occasions as community health check-ups and are thus difficult to research. The findings might suggest the need for additional support for those family caregivers, although more research with a more representative sample should be conducted.

## Conclusions

The results of this study indicate that female caregivers have a higher prevalence of high blood pressure and lowered eGFR than non-caregivers; these conditions could lead to cerebral, cardiovascular, or kidney diseases. This is one of the first reports of the possible negative association of caregiving on the objective health outcomes of families in Japan. In addition to the existing LTCI services aiming to reduce the caregiver burden, additional services are needed to assist caregivers, especially female, in order to prevent disease and improve health. More studies are needed to help develop ways to assist caregivers, since the stress of family caregiving and the associated health risks make them likely to become the next generation of care recipients.

## Abbreviations

LTCI: Long term care insurance
BMI: Body mass index
LTC: Long-term care
ADL: Activities of daily living
IADL: Instrumental activities of daily living
eGFR: Estimated glomerular filtration rate
SBP: Systolic blood pressure
DBP: Diastolic blood pressure
CKD: Chronic kidney disease
HDL-C: High density lipoprotein cholesterol
LDL-C: Low density lipoprotein cholesterol
AST: Aspartate aminotransferase
ALT: Alanine aminotransferase
γ-GTP: γ-glutamyl transpeptidase
UA: Uric acid
Cr: Creatinine
HbA1c: Glycosylated hemoglobin
CI: Confidence Interval
SES: Social economic status

## References

[1] [Status Report on the Long-term Care Insurance Projects]. Ministory of Health and Labor Welfare; 2012.

[2] Schofield HL, Bloch S, Nankervis J, Murphy B, Singh BS, Herrman HE (1999). Health and well-being of women family carers: a comparative study with a generic focus. Aust N Z J Public Health.

[3] Lokk J (2008). Caregiver strain in Parkinson’s disease and the impact of disease duration. Eur J Phys Rehabil Med.

[4] Nishimura Y (1998). Cardiovascular response relating to stress on the elderly caregivers (part1)-fosuced on caregiving act of women caregivers-. J Jpn Acad Nurs Sci.

[5] Nishimura Y (1999). Cardiovascular response relating to stress on the elderly caregivers (part2)-fosuced on night caregiving-. J Jpn Acad Nurs Sci.

[6] Okuda M, Umemura M, Yamami N, Mano Y, Hosaka T, Mizuno E (2004). A study on fatigue and health disturbance on caregivers of the elderly at home. Jpn J Prim Care.

[7] Ootsuki Y, Higuchi K (2012). A study of sense of burden experienced by family caregivers-analysis of sex difference-. J Jp Soc Psychosom Obstet Gynecol.

[8] Hirose M, Okada S, Shirasawa M (2007). Characteristics of types of cognitive caregiving appraisal by family caregivers-The related factors and coping styles-. Jpn J Gerontol.

[9] Sugiura K, Ito M, Kutsumi M, Mikami H (2010). [Gender differences in caregiving experience changes over 2-years and effects on psychological well-being of spousal caregivers in a longitudinal study]. Nihon Koshu Eisei Zasshi.

[10] Nagai K, Hori Y, Hoshino J, Hamamoto R, Suzuki Y, Sugiyama A (2011). [Subjective physical and mental health characteristics of male family caregivers]. Nihon Koshu Eisei Zasshi.

[11] Sugisawa H, Nakamura R, Nakano I, Sugisawa A (1992). [Four-year follow-up study of self-rated health and life satisfaction among caregivers]. Nihon Koshu Eisei Zasshi.

[12] Hoshino J, Hori Y, Kondo T, Maekawa A, Tamakoshi K, Sakakibara H (2009). [Physical and mental health characteristics of female caregivers]. Nihon Koshu Eisei Zasshi.

[13] Nakayama K, Morimoto K (2011). Assessment of accuracy of immediate blood separation method: a novel blood analysis strategy. Environ Health Prev Med.

[14] The Japanese Orthopaedic Association Committee for the Guidline for Locomotive Syndrome 2010 (2010). [The Guidline for Locomotive Syndrome 2010].

[15] Nakamura K (2009). Locomotive syndrome: disability-free life expectancy and locomotive organ health in a “super-aged” society. J Orthop Sci.

[16] Doi Y, Minowa M, Uchiyama M, Okawa M, Kim K, Shibui K (2000). Psychometric assessment of subjective sleep quality using the Japanese version of the Pittsburgh Sleep Quality Index (PSQI-J) in psychiatric disordered and control subjects. Psychiatry Res.

[17] Doi Y, Ogata K (2000). [Psychiatric distress and related risk factors of family caregivers who care for the demented elderly at home]. Nihon Koshu Eisei Zasshi.

[18] Furukawa TA, Kawakami N, Saitoh M, Ono Y, Nakane Y, Nakamura Y (2008). The performance of the Japanese version of the K6 and K10 in the World Mental Health Survey Japan. Int J Methods Psychiatr Res.

[19] Margolis RB, Tait RC, Krause SJ (1986). A rating system for use with patient pain drawings. Pain.

[20] Lawton MP, Brody EM (1969). Assessment of older people: self-maintaining and instrumental activities of daily living. Gerontologist.

[21] The Japanese Society of Hypertension Committee for Guidelines for the management of hypertension JSH 2009 (2009). [Guidelines for the management of hypertension JSH 2009].

[22] Japanese Society of Nephrology (2012). [Clinical Practice Guidebook for Diagnosis and Treatment of Chronic Kidney Disease].

[23] the Japan Atherosclerosis Society Committee for Guidelines for the Diagnosis and Prevention of Atherosclerotic Cardiovascular Diseases in Japan (2012). [Guidelines for the Diagnosis and Prevention of Atherosclerotic Cardiovascular Diseases in Japan 2012].

[24] the Japan Society of Ningen Dock Committee for Guideline for diagnosis criteria and health guidance after health checkup (2008). [Guideline for diagnosis criteria and health guidance after health checkup]. Ningen Dock.

[25] Japanese Society of Gout and Nucleic Acid Metabolism Committee for the Revised Guideline for the management of hyperuricemia and gout ver.2 (2012). [Guideline for the management of hyperuricemia and gout ver.2].

[26] The Committee of the Japan Diabetes Society on the Diagnostic Criteria of Diabetes Mellitus (2010). [Report of the Committee on the Classification and Diagnostic Criteria of Diabetes Mellitus]. J Jpn Diabetes Soc.

[27] Oertelt-Prigione S, Regitz-Zagrosek V (2012). Sex and Gender Aspects in Clinical Medicine.

[28] Noumi M, Ishizaki A, Isobe M, Ogata I (2011). [Introduction of Safe and Simple Method of Blood Collection for the Life Insurance Examination-Comparison with Intravenous Blood Collection Method-]. J Assoc Insur Med Jpn.

[29] Capistrant BD, Moon JR, Glymour MM (2012). Spousal caregiving and incident hypertension. Am J Hypertens.

[30] Zimmerman RS, Frohlich ED (1990). Stress and hypertension. J Hypertens Suppl.

[31] Pickering TG (1997). The effects of environmental and lifestyle factors on blood pressure and the intermediary role of the sympathetic nervous system. J Hum Hypertens.

[32] von Kanel R, Mausbach BT, Dimsdale JE, Mills PJ, Patterson TL, Ancoli-Israel S (2012). Effect of chronic dementia caregiving and major transitions in the caregiving situation on kidney function: a longitudinal study. Psychosom Med.

[33] Dukkipati R, Adler S, Mehrotra R (2008). Cardiovascular implications of chronic kidney disease in older adults. Drugs Aging.

[34] Bruce MA, Beech BM, Sims M, Brown TN, Wyatt SB, Taylor HA (2009). Social environmental stressors, psychological factors, and kidney disease. J Investig Med.

[35] Kokubo Y, Nakamura S, Okamura T, Yoshimasa Y, Makino H, Watanabe M (2009). Relationship between blood pressure category and incidence of stroke and myocardial infarction in an urban Japanese population with and without chronic kidney disease: the Suita Study. Stroke.

[36] [Outline of the National Health and Nutrition Survey Japan, 2010]. Ministry of Healh, Labor and Welfare; 2010.

[37] Del-Pino-Casado R, Frias-Osuna A, Palomino-Moral PA, Ramon Martinez-Riera J (2012). Gender differences regarding informal caregivers of older people. J Nurs Scholarsh.

[38] Poysti MM, Laakkonen ML, Strandberg T, Savikko N, Tilvis RS, Eloniemi-Sulkava U (2012). Gender differences in dementia spousal caregiving. Int J Alzheimers Dis.

